# Apocynin and ebselen reduce influenza A virus-induced lung inflammation in cigarette smoke-exposed mice

**DOI:** 10.1038/srep20983

**Published:** 2016-02-15

**Authors:** L. C. Oostwoud, P. Gunasinghe, H. J. Seow, J. M. Ye, S. Selemidis, S. Bozinovski, R. Vlahos

**Affiliations:** 1 Lung Health Research Centre, Department of Pharmacology & Therapeutics, The University of Melbourne, Victoria, Australia; 2 Department of Molecular Pharmacology, The University of Groningen, Groningen, The Netherlands; 3 School of Health and Biomedical Sciences, RMIT University, Bundoora, Victoria, Australia; 4 Department of Pharmacology, Monash University, Clayton, Victoria, Australia

## Abstract

Influenza A virus (IAV) infections are a common cause of acute exacerbations of chronic obstructive pulmonary disease (AECOPD). Oxidative stress is increased in COPD, IAV-induced lung inflammation and AECOPD. Therefore, we investigated whether targeting oxidative stress with the Nox2 oxidase inhibitors and ROS scavengers, apocynin and ebselen could ameliorate lung inflammation in a mouse model of AECOPD. Male BALB/c mice were exposed to cigarette smoke (CS) generated from 9 cigarettes per day for 4 days. On day 5, mice were infected with 1 × 10^4.5^ PFUs of the IAV Mem71 (H3N1). BALF inflammation, viral titers, superoxide production and whole lung cytokine, chemokine and protease mRNA expression were assessed 3 and 7 days post infection. IAV infection resulted in a greater increase in BALF inflammation in mice that had been exposed to CS compared to non-smoking mice. This increase in BALF inflammation in CS-exposed mice caused by IAV infection was associated with elevated gene expression of pro-inflammatory cytokines, chemokines and proteases, compared to CS alone mice. Apocynin and ebselen significantly reduced the exacerbated BALF inflammation and pro-inflammatory cytokine, chemokine and protease expression caused by IAV infection in CS mice. Targeting oxidative stress using apocynin and ebselen reduces IAV-induced lung inflammation in CS-exposed mice and may be therapeutically exploited to alleviate AECOPD.

Chronic Obstructive Pulmonary Disease (COPD) is a major incurable disease and is the 4th leading cause of death worldwide^1^. COPD is a “disease characterized by airflow limitation that is not fully reversible. The airflow limitation is usually progressive and associated with an abnormal inflammatory response of lungs to noxious particles and gases”^2^. Cigarette smoking is the major cause of COPD and accounts for more than 95% of cases in industrialized countries^3^, but other environmental pollutants are important causes in developing countries^4^. In addition, increased oxidative stress plays a key role in the progression of the inflammatory process^5^. The abnormal inflammatory response in the lungs of COPD patients consists of an influx of macrophages, neutrophils, T lymphocytes and increased levels of pro-inflammatory cytokines (e.g. tumour necrosis factor-α [TNF-α], interleukin-8 [IL-8], granulocyte-macrophage colony stimulating factor [GM-CSF])^6^, chemokines (e.g. monocyte chemoattractant protein-1 [MCP-1] and matrix metalloproteinases (e.g. MMP-1, -9 and -12)^6^. Current pharmacological therapies do not effectively relieve the symptoms, inflammation and decline in lung function in COPD^6^.

Importantly, patients with COPD are susceptible to acute exacerbations of COPD (AECOPD), defined as “a sustained worsening of patient’s condition from the stable state and beyond normal day-day variations that is acute in onset and necessitates a change in regular medication in a patient with underlying COPD”^7^. Exacerbations are a common occurrence in COPD patients and contribute mainly to morbidity, mortality and reduced health^7^. During AECOPD, there are increased levels of neutrophils and macrophages and up-regulation of IL-6, IL-8, TNF-α, interferon gamma (IFN)-γ, GM-CSF and MMP-12 in sputum^7^,^8^. Moreover, there is increased activation of nuclear factor–ĸB (NF-ĸB) in alveolar macrophages, and expression of inflammatory markers such as TNF-α and IL-8^6^,^8^. AECOPD are due to a number of aetiological factors, predominantly viral and bacterial infections with 40–60% attributed to viral infections^7^. The majority of these infections are due to respiratory syncytial virus (RSV) (22%), influenza A (25%) and rhinovirus (36%)^9^. Even though RSV is the most commonly isolated virus in exacerbations, influenza has the potential to be more problematic due to the likelihood of epidemics and pandemics^7^.

Previous studies have shown that prior cigarette smoke (CS) exposure exacerbates lung inflammation and disrupts the resolution of influenza infection in mice^10^,^11^. Current treatments to combat influenza outbreaks are primarily focused on targeting the mechanisms of viral infection and replication. Strain-specific vaccines and anti-viral drugs, such as Relenza and Tamiflu, have some efficacy in the treatment and/or prevention of influenza infection^12^,^13^. However, evidence suggests that current treatments are problematic due to the ability of influenza to develop resistance against anti-viral therapeutics^14^. In addition, the effectiveness of vaccination depends on patient’s immune status, age, and co-morbidities^15^.

Oxidative stress, which is defined as the persistent overproduction of reactive oxygen species (ROS) that overwhelms endogenous antioxidant defence systems, has been shown to play a role in COPD, AECOPD and influenza A virus (IAV)-induced lung inflammation and damage^12^,^16^,^17^,^18^. There is an increased oxidant burden in smokers resulting from the fact that CS itself contains over 4700 different chemical compounds and more than 10^15^ oxidants/free radicals per puff^16^,^19^,^20^,^21^. These oxidants can give rise to additional ROS generated enzymatically by inflammatory and epithelial cells within the lung as part of an inflammatory-immune response towards a pathogen or irritant. Activation of macrophages and neutrophils by CS generates superoxide radicals (O_2_^●^^−^), which can then either react with nitric oxide (NO) to form reactive peroxynitrite molecules (ONOO^−^) or alternatively be rapidly converted to damaging hydrogen peroxide (H_2_O_2_) under the influence of superoxide dismutase (SOD). This in turn can result in the non-enzymatic production of the more damaging hydroxyl radical (^●^OH) from H_2_O_2_ in the presence of Fe^2+^ through the Fenton reaction. Nicotinamide adenine dinucleotide phosphate (NADPH) oxidases are the major enzymatic sources of superoxide production by inflammatory cells^13^,^22^,^23^.

Increased levels of ROS have been implicated in initiating inflammatory responses in the lungs through the activation of transcriptional factors such as NF-κB and activator protein-1 (AP-1), signal transduction pathways, chromatin remodelling and gene expression of pro-inflammatory mediators^16^,^17^,^24^. Oxidative stress can cause deleterious effects in the body such as DNA damage, lipid peroxidation and protein denaturation^25^. Moreover, oxidative stress can affect remodelling of extracellular matrix, mitochondrial respiration, and protective mechanisms of the lung such as the surfactants to increase the strength of alveoli^16^. During COPD exacerbations, the newly recruited neutrophils activate oxidant-sensitive transcription factors leading to increased transcription of pro-inflammatory genes resulting in oxidative burst^26^. Moreover, there is an increased concentration of H_2_O_2_ in exhaled breath condensate (EBC) of COPD patients and this is further elevated during AECOPD^3^,^26^. H_2_O_2_ can activate NF-κB resulting in pro-inflammatory gene activation, thereby worsening the condition^27^,^28^. The glutathione system is the major anti-oxidant mechanism in the airways and is upregulated in response to CS^3^. However, during severe COPD exacerbations anti-oxidant enzymes such as glutathione peroxidase (GPx) are depleted leading to increased oxidative stress^3^,^26^. We have previously shown that GPx-1 deficient mice exposed to CS have enhanced BALF inflammation suggesting GPx-1 has a protective role against CS-induced lung inflammation^29^. More recently, we showed that GPx-1 protects against IAV-induced lung inflammation^30^. The GPx mimetic ebselen has been shown to reduce sephadex-31, LPS-32 and CS-induced lung inflammation^29^. We have also shown that mice deficient in Nox2 had markedly reduced superoxide radicals production and lung inflammation following IAV infection^18^. Apocynin, an NADPH oxidase-2 inhibitor, significantly reduced IAV-induced lung inflammation, superoxide production and viral titres^18^. Stefanska *et al.* have recently shown that apocynin treatment reduced the H_2_O_2_ concentrations in EBC of COPD patients in comparison to the placebo group^33^. Moreover, apocynin inhalation decreased the concentrations of nitrite in airways of COPD patients, implying apocynin could be considered as an anti-inflammatory agent in COPD^34^.

Therefore, given that there is increased oxidative stress in AECOPD, and that ROS such as superoxide and H_2_O_2_ are elevated in human AECOPD, we investigated whether targeting the production of ROS with apocynin (a small molecule that inhibits NADPH oxidase assembly) and ebselen (an active organo-selenium compound that mimics the action of GPx) can reduce lung inflammation in an animal model of AECOPD.

## Methods

### Animals

Specific pathogen-free BALB/c mice aged 7–9 weeks and weighing ~20 g were obtained from the Animal Resource Centre Pty Ltd (Perth, Australia). The animals were housed at 20 °C on a 12-h day/night cycle in sterile micro-isolators and fed a standard sterile diet of Purina mouse chow with water allowed *ad libitum*. The experiments described in this manuscript were approved by the Animal Ethics Committee of The University of Melbourne (Application ID 1112185) and conducted in compliance with the guidelines of the National Health and Medical Research Council of Australia on animal experimentation.

### Cigarette smoke exposure

Mice were placed in an 18-L perspex chamber (The Plastic Man, Huntingdale, Victoria, Australia) in a class II biosafety cabinet (AES Environmental Pty Ltd, Melbourne, Victoria, Australia) and exposed to CS generated from 9 cigarettes per day for 4 days as previously described^35^,^36^. Briefly, mice were exposed to CS generated from 9 cigarettes/day for 4 days, delivered three times per day at 9 AM, 12 noon and 3 PM with 3 cigarettes spaced over 1 h. Smoke was generated in 50-ml tidal volumes over 10 s, by use of timed draw-back mimicking normal smoking inhalation volume and cigarette burn rate. The mean total suspended particulate mass concentration in the chamber containing CS was ~420 mg m^−3^. Sham-exposed mice were placed in an 18-L perspex chamber but did not receive CS. Commercially available filter-tipped Winfield Red cigarettes (manufactured by Philip Morris, Australia) of the following composition were used: 16 mg or less of tar, 1.2 mg or less of nicotine, and 15 mg or less of CO.

### Influenza A virus infection

After 4 days of CS or sham exposure, mice were anaesthetized by placing them in a small plastic container with cotton gauze onto which 2 ml of methoxyflurane (Medical Developments International Ltd, Australia) had been applied. Anaesthetized mice were infected intranasally with 1 × 10^4.5^ plaque forming units (PFU) of the intermediate virulent IAV Mem71 (H3N1) in a 30 μl volume, diluted in PBS. Control animals were given 30 μl of PBS + VP-SFM vehicle (diluent). Mice were then culled 3 and 7 days (d3 and d7) after viral infection. The time points of 3 and 7 days post infection are based on our previous studies and represent the peak and resolution (respectively) of influenza infection^10^. There was no further CS exposure after IAV infection. Mouse body weight, food consumption and the well-being of mice were monitored daily at approximately the same time, to ensure that mice were not going through severe distress in response to CS and IAV infection. Food consumption was calculated by measuring the amount of food remaining in cages every 24 h and dividing that by the number of mice in the cages.

### Ebselen and apocynin treatment

Mice were treated once a day with ebselen (10 mg kg^−1^) or vehicle (5% carboxymethyl [CM]-cellulose made up in distilled water) via oral gavage 3 h before IAV infection and daily thereafter until they were killed 3 and 7 days post infection. In separate experiments, mice were treated with apocynin (5 mg kg^−1^) or vehicle (0.1% Dimethyl Sulfoxide [DMSO] in PBS) administered via intraperitoneal injection (i.p.) 3 h prior to IAV infection and daily thereafter until they were killed 3 and 7 days post infection. The doses for both apocynin and ebselen are based on our previous publications^18^,^29^,^30^.

### Bronchoalveolar lavage and lung collection

Animals were culled by i.p. injection of sodium pentobarbitone (360 mg kg^−1^) (Sigma Aldrich, St. Louis, MO) on day 3 or day 7 post infection. Lungs were then lavaged *in situ* with a 400 μl aliquot of PBS, followed by three 300 μl aliquots as previously described^35^,^36^. In total up to 1 ml of bronchoalvealor lavage fluid (BALF) was retrieved per mouse. The total number of viable cells in the BALF was determined, cytospins were prepared using 50–200 μl of BALF, and cells were differentiated by standard morphological criteria. Residual BALF was centrifuged to collect the supernatant for storage at −80 °C. Whole lungs were perfused free of blood via right ventricular perfusion with 5 ml of PBS, rapidly excised en bloc, rinsed in PBS, blotted, snap frozen in liquid nitrogen and stored at −80 °C.

### RNA extraction and quantitative real-time PCR

Total RNA was extracted from approximately 15 mg of whole lung tissue pooled from five to eight mice per treatment group using RNeasy Mini Kits (Qiagen, Hilden Germany), reverse transcribed with High Capacity RNA-to-cDNA kit (Life Technologies, Carlsbad, CA), and duplicate real-time PCR reactions with Life Technologies pre-developed Taqman assay reagents were performed. 18 S rRNA was used as the internal control as previously described^35^,^36^. The threshold cycle (*C*_T_) value is the PCR cycle number (out of 40) at which the measured fluorescent signal exceeds a calculated background threshold identifying amplification of the target sequence value and is proportional to the number of input target copies present in the sample. *C*_T_ numbers were transformed with the ΔΔ*C*_T_ (threshold cycle time) and relative value method and were expressed relative to 18S rRNA levels.

### Lung homogenization and virus titrations

Lungs from terminally anesthetized IAV-infected mice were removed, rinsed in PBS, weighed, and homogenized in 1 ml of Dulbecco’s Modified Eagle’s Medium (DMEM) (Life Technologies, Carlsbad, CA). Clarified homogenate was snap frozen and stored at −80 °C until required. Virus was quantitated by plaque assay in MDCK cells as previously published^10^,^18^,^30^.

### Superoxide detection with L-O12 enhanced chemiluminescence

BALF inflammatory cells were exposed to the chemiluminescent probe, L-O12 (100 mM; Wako Laboratories, Japan) in the absence (for basal measurements) or presence of the PKC and NADPH oxidase activator, phorbol 12, 13 dibutyrate (PDBu, 1 mM; Sigma) and dispensed into 96 well white opti-plates for luminescence reading with the TopCount (Perkin Elmer Packard). Photon emission was recorded from each well every 2 min and averaged over 45 min. Individual data points for each group were derived from the average of 2 replicates, subtracted against the average background values and normalized for total cell numbers and expressed as relative light units (RLU). We have previously shown incubation of cells with superoxide dismutase (SOD; 600 U/ml) to inactivate superoxide almost abolishes the chemiluminescence signal verifying that the L-O12 chemiluminescence signal was due to superoxide^18^.

### Statistical analysis

Data are presented as grouped data expressed as mean ± standard error of the mean (SEM); *n* represents the number of mice per treatment group. Differences were determined by two-way analysis of variance (ANOVA) followed by Bonferroni *post hoc* tests for multiple comparisons. In some cases, Student’s unpaired *t*-test was used to determine if there were significant differences between means of pairs. All statistical analyses were performed using GraphPad Prism for Windows (Version 5.03). Statistical significance was indicated by using *P* < 0.05.

## Results

### Apocynin reduces BALF inflammation in cigarette smoke exposed and influenza A virus-infected mice

**Figure 1:**
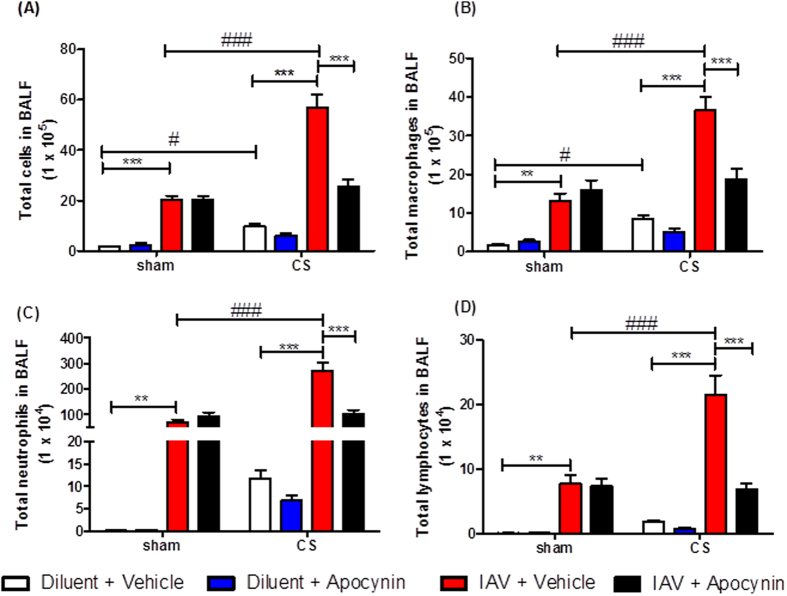
The effect of 3 day apocynin (5 mg kg^−1^) administration on BALF cells in mice after exposure to cigarette smoke (CS) and infection with influenza A virus (IAV). BALF cellularity is shown as (**A**) the total number of cells, (**B**) macrophages, (**C**) neutrophils and (**D**) lymphocytes. Data are expressed as mean ± SEM for n = 5–8 per treatment group. Two-way ANOVA with Bonferroni post-hoc test was performed to assess statistical significance. **P < 0.01, ***P < 0.001, ^#^*P* < 0.05, ^###^*P* < 0.001.

Mice exposed to CS and treated with diluent had a small increase in BALF total cell number, macrophages and neutrophils when compared to sham + diluent mice at day 3 (Fig. 1). Sham mice treated with IAV + vehicle had significantly more BALF total cells, macrophages, neutrophils and lymphocytes compared to sham + diluent + vehicle mice 3 days post infection (Fig. 1). However, CS-exposed mice treated with IAV had significantly more BALF total cells, macrophages, neutrophils and lymphocytes when compared to sham + IAV + vehicle mice (Fig. 1) (n = 5–8, *P* < 0.05). Administration of apocynin (5 mg kg^−1^) for 3 days caused a significant decrease in BALF total cells, macrophages, neutrophils and lymphocytes in CS + IAV mice compared to CS + IAV + vehicle mice (Fig. 1) (n = 6–8, *P* < 0.05).

**Figure 2:**
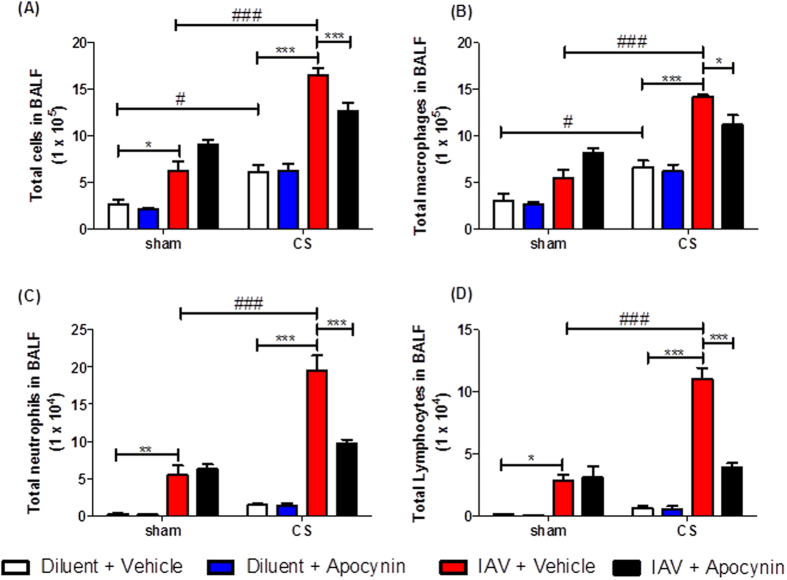
The effect of 7 day apocynin (5 mg kg^−1^) administration on BALF cells in mice after exposure to cigarette smoke (CS) and infection with influenza A virus (IAV). BALF cellularity is shown as (**A**) the total number of cells, (**B**) macrophages, (**C**) neutrophils and (**D**) lymphocytes. Data are expressed as mean ± SEM for n = 5–8 mice per treatment group. Two-way ANOVA with Bonferroni post-hoc test was performed to assess statistical significance. *P < 0.05, **P < 0.01, ***P < 0.001, ^#^*P* < 0.05, ^###^*P* < 0.001.

As in the d3 experiment, d7 CS + IAV mice had significantly more BALF total cells, macrophages, neutrophils and lymphocytes when compared to sham + IAV + vehicle mice (Fig. 2) (n = 5–8, *P* < 0.05). Administration of apocynin (5 mg kg^−1^) for 7 days caused a significant decrease in BALF total cells, macrophages, neutrophils and lymphocytes in CS + IAV mice compared to CS + IAV + vehicle mice (Fig. 2) (n = 6–8, P < 0.05). However on d7 cell counts were lower than on d3 and therefore show that resolution of inflammation has occurred during these days.

### Effect of apocynin on chemokine, cytokine and protease mRNA expression in cigarette smoke exposed and influenza A virus-infected mice

To identify which mediators drive the enhanced lung inflammation in response to CS and IAV infection, mRNA levels of a panel of inflammatory chemokines, cytokines and proteases implicated in COPD and IAV infection were measured in whole lung by Q-PCR at both 3 and 7 days post infection.

Three days post infection, CS + IAV mice had increased mRNA expression of pro-inflammatory chemokines (CCL-2, CXCL-2), cytokines (GM-CSF, TNF-α, IL-1β, IL-6) and proteases (MMP-12) compared to sham + IAV + diluent mice (Table 1). However, CS + IAV mice treated with apocynin for 3 days had markedly reduced mRNA expression of chemokines (CCL-2, CXCL-2), cytokines (GM-CSF, TNF-α, IL-1β, IL-6) and proteases (MMP-12) compared to CS + IAV + vehicle mice (Table 1).

Seven days post infection, CS + IAV mice had increased mRNA expression of pro-inflammatory chemokines (CCL-2, CXCL-2), cytokines (GM-CSF, TNF-α, IL-1β, IL-6) and proteases (MMP-12) compared to sham + IAV + diluent mice (Table 2). However, CS + IAV mice treated with apocynin 7 days had markedly reduced mRNA expression of chemokines (CCL-2, CXCL-2), cytokines (GM-CSF, TNF-α, IL-1β, IL-6) and proteases (MMP-12) compared to CS + IAV + vehicle mice (Table 2).

### Effect of apocynin on viral load in cigarette smoke exposed and influenza A virus-infected mice

**Figure 3:**
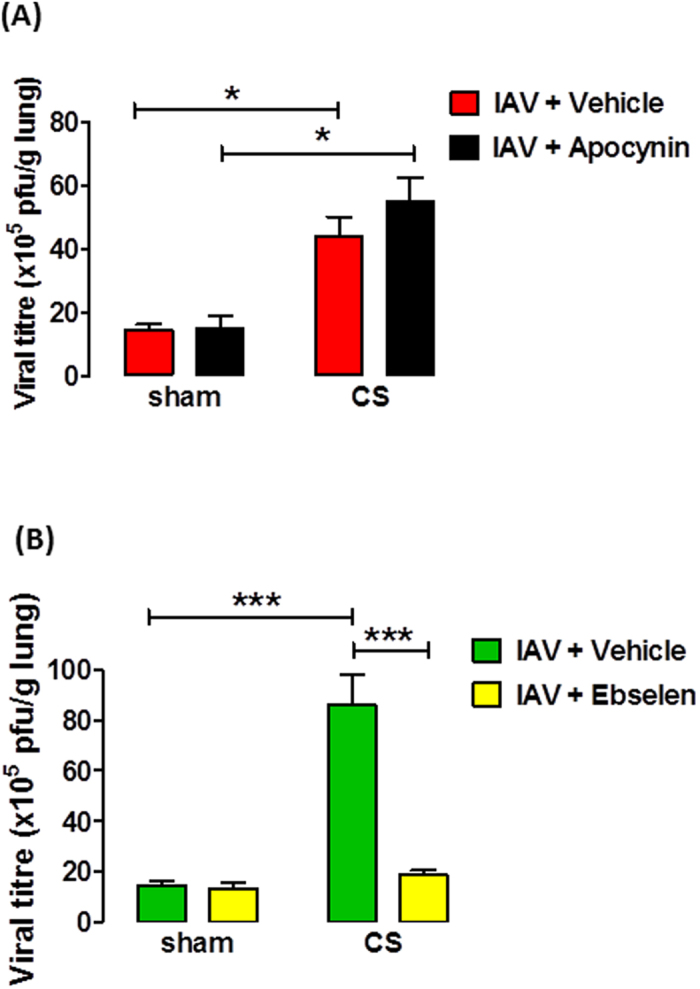
Effect of apocynin (**A**) and ebselen (**B**) on lung viral titres in mice exposed to cigarette smoke (CS) and infected with influenza A virus (IAV). Lung viral titres were determined by plaque assays on individual lung homogenates from IAV-infected mice. Data in plaque forming units (PFU) per gram of lung are expressed as mean ± SEM for n = 4–6 mice, 3 days post infection. Two-way ANOVA with Bonferroni post-hoc test was performed to assess statistical significance. *P < 0.05, ***P < 0.001.

As seen in Fig. 3A, there was a considerable amount of virus present in sham + IAV and smoke + IAV mice at d3. However, apocynin (5 mg kg^−1^) treatment had no effect on viral titres in either IAV-treated nor CS + IAV mice (n = 6, *P* > 0.05).

### Apocynin reduces superoxide production in mice exposed to cigarette smoke and infected with influenza A virus

**Figure 4:**
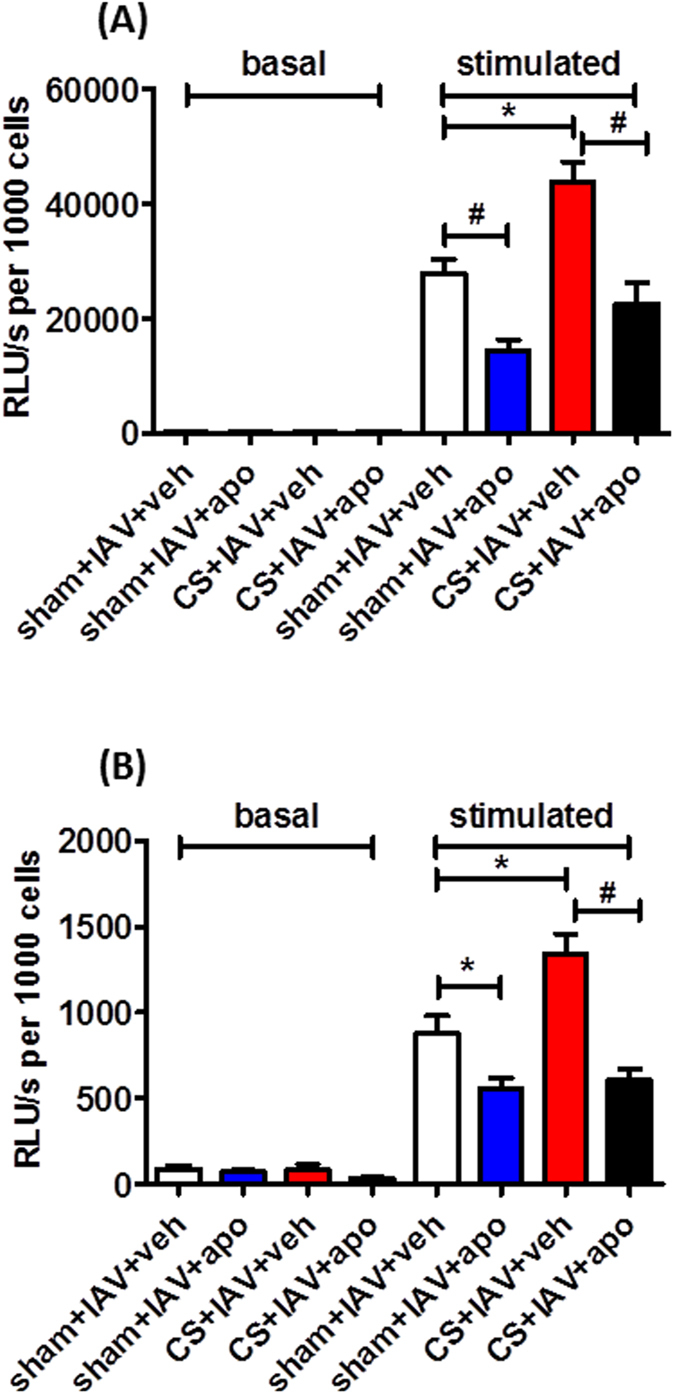
Effect of apocynin on superoxide radical production from BALF cells obtained from mice exposed to cigarette smoke (CS) and infected with influenza A virus (IAV). BALF cells recovered from apocynin (5 mg kg^−1^) or vehicle (0.1% DMSO) treated CS-exposed and IAV-infected mice 3 (**A**) and 7 (**B**) days post infection were assessed *ex vivo* for superoxide production under basal and PDBu-stimulated conditions. Data in relative light units(RLU)/second per 1000 cells are expressed as mean ± SEM for n = 9–12 mice. Statistical significance was assessed using two-way ANOVA and Bonferroni post-hoc test. *P < 0.05, ^#^*P* < 0.001.

Basal levels of superoxide were similar for all treatment groups. BAL cells isolated from d3 IAV-infected mice showed a significant increase in phorbol 12, 13 dibutyrate (PDBu)-stimulated superoxide production compared to basal (Fig. 4A) (n = 5–6, *P* < 0.001). In addition, BAL cells isolated from CS-exposed mice infected with IAV had even greater PDBu-stimulated superoxide production compared to IAV-infected mice. Apocynin (5 mg kg^−1^) treatment caused a significant reduction in PDBu-stimulated superoxide in IAV-infected mice and CS + IAV mice (Fig. 4A) (n = 9–12, *P* < 0.001).

As with the d3 IAV-infected mice, d7 IAV-infected mice showed a significant increase in PDBu-stimulated superoxide production compared to basal (Fig. 4B) (n = 9–12, *P* < 0.001). In addition, CS-exposed mice infected with IAV had even greater PDBu-stimulated superoxide production compared to IAV-infected mice. Apocynin (5 mg kg^−1^) treatment caused a significant reduction in PDBu-stimulated superoxide in IAV-infected mice and CS + IAV mice (Fig. 4B) (n = 9–12, *P* < 0.001). However d7 PDBu-stimulated superoxide production was lower than that observed in d3 mice. Basal levels of superoxide were similar for all treatment groups.

### Effects of apocynin on body weight and food consumption in cigarette smoke exposed and influenza A virus-infected mice

**Figure 5:**
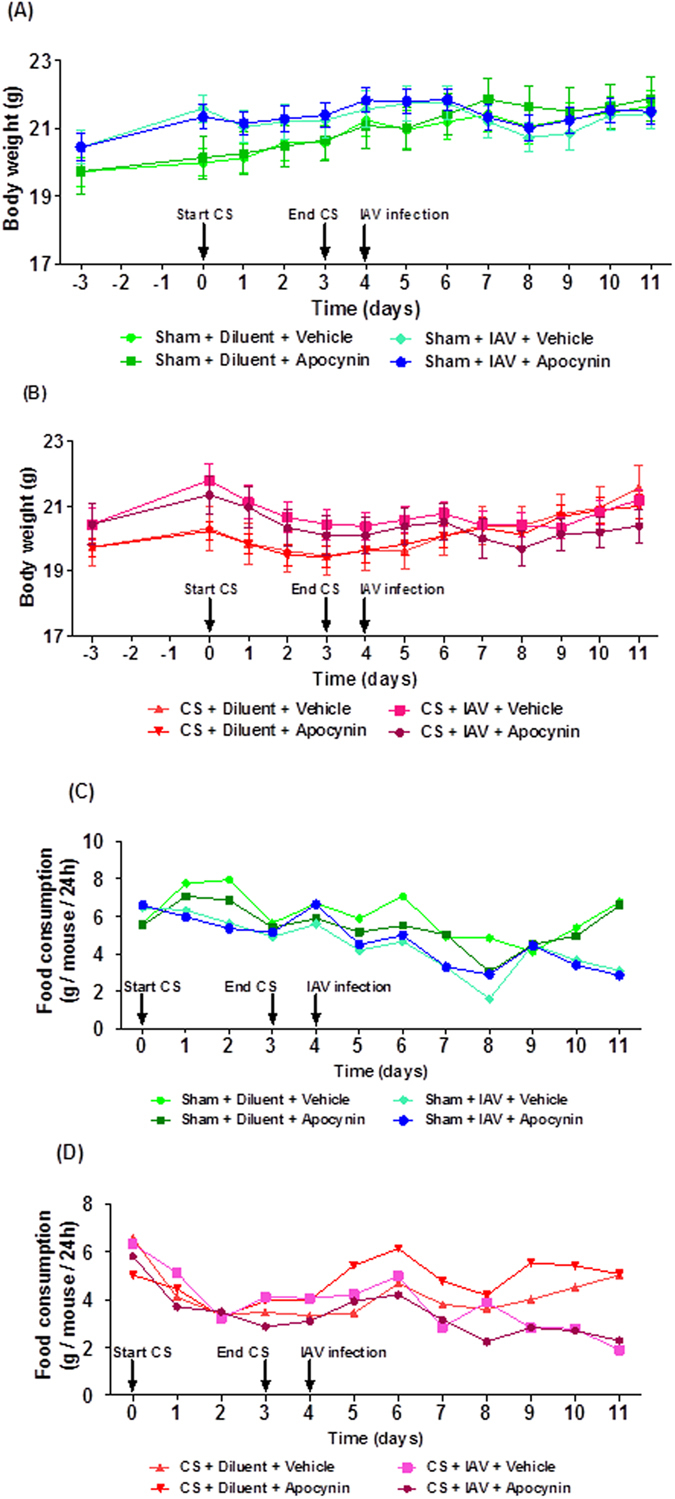
Effect of apocynin on (**A,B**) body weight and (**C,D**) food consumption in mice exposed to cigarette smoke (CS) and infected with influenza A virus (IAV). Mice (n = 5–8) were weight matched into different treatment groups on day −3 and then the body weight of each mouse was recorded daily throughout the experimental protocol until 7 days post infection (d7). Mice were treated with apocynin (5 mg kg^−1^) or vehicle (0.1% DMSO in PBS) i.p. 3 h before infection with 1 × 10^4.5^ PFU of Mem71 virus and then daily thereafter. Food consumption was calculated by measuring the amount of food remaining in cages every 24 h and dividing that by the number of mice in the cages.

Mice exposed to CS for 4 days lost weight compared to sham-exposed mice (Fig. 5A,B). Apocynin (5 mg kg^−1^) treatment did not affect CS-induced weight loss. IAV infection caused a small amount of weight loss at days 3–5 post-infection compared to sham + diluent mice, but this was not statistically significant (*P* > 0.05). In addition, IAV infection after CS exposure did not cause further significant weight loss compared to CS + diluent mice. Once again, apocynin (5 mg kg^−1^) treatment had no impact on body weight in sham + IAV mice and CS + IAV mice.

In general, the food consumption by CS-exposed mice was lower than the sham-exposed mice over the four-day smoking period (Fig. 5C,D). In addition, IAV infection didn’t impact on food intake nor did treatment with apocynin.

### Ebselen reduces BALF inflammation in cigarette smoke exposed and influenza A virus-infected mice

**Figure 6:**
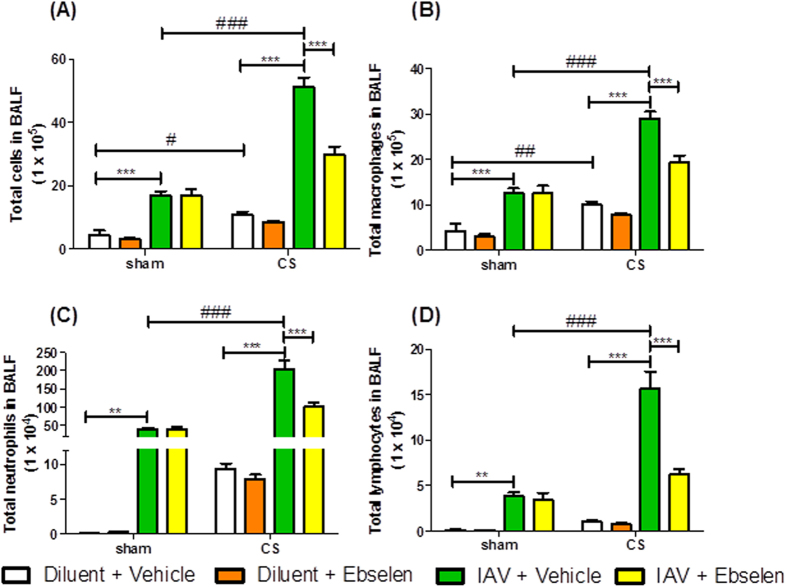
The effect of 3 day ebselen (10 mg kg^−1^) administration on BALF cells in mice after exposure to cigarette smoke (CS) and infection with influenza A virus (IAV). BALF cellularity is shown as (**A**) the total number of cells, (**B**) macrophages, (**C**) neutrophils and (**D**) lymphocytes. Data are expressed as mean ± SEM for n = 6–8 mice per treatment group. Two-way ANOVA with Bonferroni post-hoc test was performed to assess statistical significance. *P < 0.05, ***P < 0.001, ^#^*P* < 0.05, ^##^*P* < 0.01, ^###^*P* < 0.001.

Mice exposed to CS and treated with diluent had a small increase in BALF total cell number, macrophages and neutrophils when compared to sham + diluent mice at day 3 (Fig. 6A–D). Sham mice treated with IAV + vehicle had significantly more BALF total cells, macrophages, neutrophils and lymphocytes compared to sham + diluent + vehicle mice 3 days post infection (Fig. 6A–D, n = 6, *P* < 0.05). However, CS-exposed mice treated with IAV had significantly more BALF total cells, macrophages, neutrophils and lymphocytes when compared to sham + IAV + vehicle mice (Fig. 6A–D, n = 6, *P* < 0.05). Administration of ebselen (10 mg kg^−1^) for 3 days caused a significant decrease in BALF total cells, macrophages, neutrophils and lymphocytes in CS + IAV mice compared to CS + IAV + vehicle mice (Fig. 6A–D, n = 6–8, *P* < 0.05).

**Figure 7:**
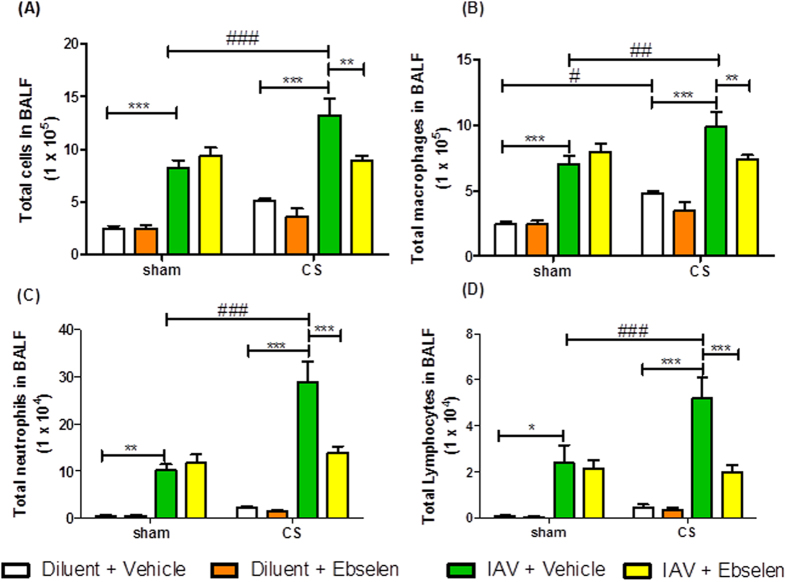
The effect of 7 day ebselen (10 mg kg^−1^) administration on BALF cells in mice after exposure to cigarette smoke (CS) and infection with influenza A virus (IAV). BALF cellularity is shown as (**A**) the total number of cells, (**B**) macrophages, (**C**) neutrophils and (**D**) lymphocytes. Data are expressed as mean ± SEM for n = 6–8 mice per treatment group. Two-way ANOVA with Bonferroni post-hoc test was performed to assess statistical significance. *P < 0.05, **P < 0.01, ***P < 0.001, ^#^*P* < 0.05, ^##^*P* < 0.01, ^###^*P* < 0.001.

As in the d3 experiment, d7 CS + IAV mice had significantly more BALF total cells, macrophages, neutrophils and lymphocytes when compared to sham + IAV + vehicle mice (Fig. 7A–D, n = 6, *P* < 0.05). Administration of ebselen (10 mg kg^−1^) for 7 days caused a significant decrease in BALF total cells, macrophages, neutrophils and lymphocytes in CS + IAV mice compared to CS + IAV + vehicle mice (Fig. 7A–D, n = 6–8, *P* < 0.05). However d7 cell counts were lower than on d3 and therefore show that resolution of inflammation has occurred during these days.

### Effect of ebselen administration on chemokines, cytokines and protease mRNA expression in cigarette smoke exposed and influenza A virus-infected mice

Since we had previously identified a panel of inflammatory genes that were markedly increased in the lungs of mice treated with the combination of CS and IAV, we next assessed whether ebselen had an inhibitory effect on their expression.

Three days post infection, sham + IAV + vehicle mice had increased mRNA expression of pro-inflammatory chemokines (CCL-2, CXCL-2, CXCL-10), cytokines (GM-CSF, TNF-α, IL-1β, IL-6) and proteases (MMP-12) compared to sham + diluent + vehicle mice. However, IAV mice treated with ebselen had markedly lower mRNA expression of chemokines (CCL-2, CXCL-2, CXCL-10), cytokines (GM-CSF, TNF-α, IL-1β, IL-6) and proteases (MMP-12) compared to IAV + vehicle mice (Table 3). CS + IAV + ebselen mice had reduced GM-CSF, IL-6 and MMP-12 but surprisingly increased TNF-α, IL-1β, CCL-2, CXCL-2 and CXCL-10 compared to CS + IAV + vehicle mice (Table 3).

Seven days post infection, CS + IAV mice had increased mRNA expression of pro-inflammatory chemokines (CCL-2, CXCL-2, CXCL-9, CXCL-10), cytokines (GM-CSF, TNF-α, IL-1β, IL-6) and proteases (MMP-12) compared to sham + IAV + diluent mice or CS + diluent mice (Table 4). However, CS + IAV mice treated with ebselen for 7 days had markedly reduced mRNA expression of chemokines (CCL-2, CXCL-2, CXCL-9, CXCL-10), cytokines (GM-CSF, TNF-α, IL-1β, IL-6) and proteases (MMP-12) compared to CS + IAV + vehicle mice (Table 4).

### Clearance of influenza A virus by ebselen following cigarette smoke exposure and influenza A virus infection

Plaque assays on lung homogenates were used to determine the amount of virus in the lungs of mice infected with IAV. As seen in Fig. 3B, there was a considerable amount of virus present in sham + IAV mice 3 days post infection. Moreover, CS exposure caused a significant increase in viral titre compared to sham + IAV mice (n = 4–6, *P* < 0.05) and ebselen caused a significant decrease in the amount of virus in CS + IAV mice (n = 4–6, *P* < 0.05). In contrast, there was very little virus present in the lungs of sham + IAV mice 7 days post infection and this was comparable to CS + IAV mice (n = 6, data not shown).

### Effect of ebselen on body weights and food consumption in cigarette smoke exposed and influenza A virus-infected mice

**Figure 8:**
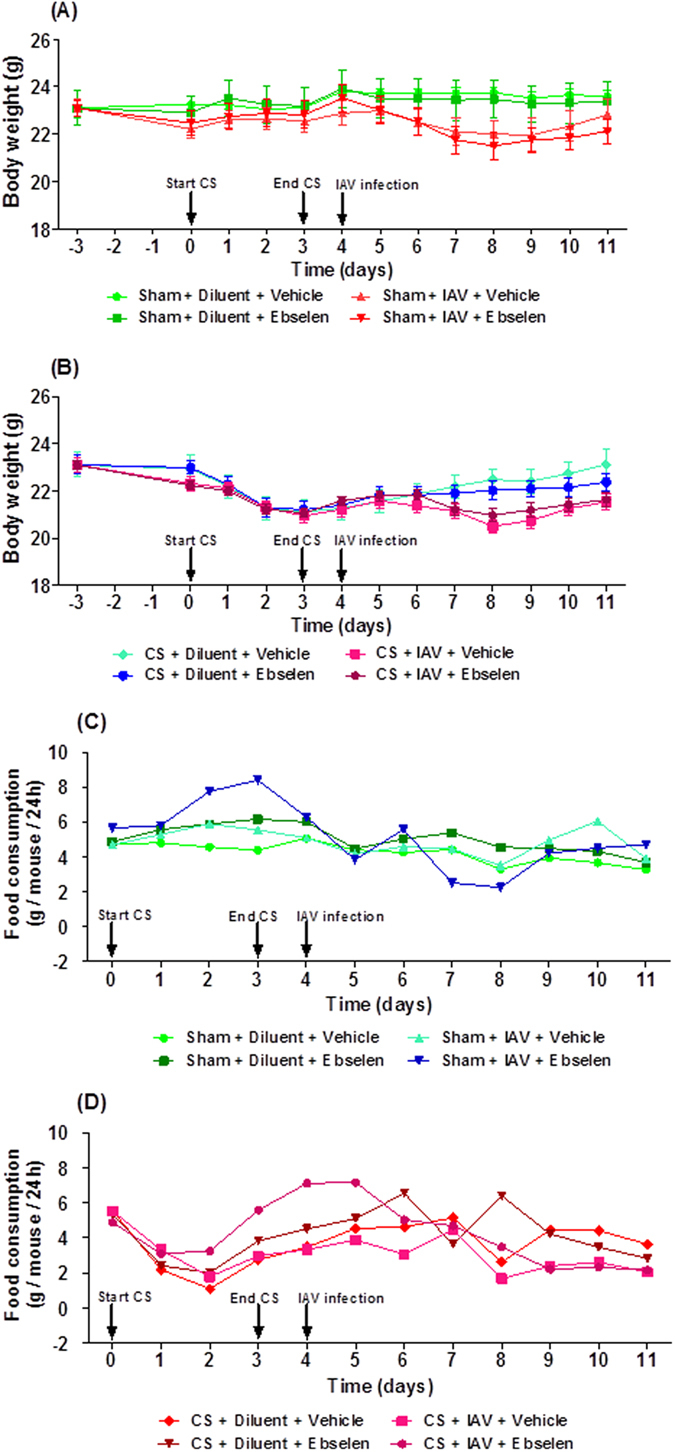
Effect of ebselen on (**A,B**) body weight and (**C,D**) food consumption in mice exposed to cigarette smoke (CS) and infected with influenza A virus (IAV). Mice (n = 6–8) were weight matched into different treatment groups on day −3 and then the body weight of each mouse was recorded daily throughout the experimental protocol until 7 days post infection (d7). Mice were treated with ebselen (10 mg kg^−1^) or vehicle (5% CM-cellulose) by oral gavage 3 h before infection with 1 × 10^4.5^ PFU of Mem71 virus and then daily thereafter. Food consumption was calculated by measuring the amount of food remaining in cages every 24 h and dividing that by the number of mice in the cages.

Mice exposed to CS for 4 days lost weight compared to sham-exposed mice (Fig. 8). Ebselen (10 mg kg^−1^) treatment did not affect CS-induced weight loss (*P* > 0.05). IAV infection caused a small amount of weight loss at days 3–6 post infection compared to sham + diluent mice, but this was not statistically significant (*P* > 0.05). However, it appeared that CS + IAV mice weighed less than CS alone mice on days 4–6 post infection. Once again, ebselen (10 mg kg^−1^) treatment had no impact on body weight in sham + IAV mice and CS + IAV mice.

In general, the food consumption by CS-exposed mice was lower than the sham-exposed mice over the four-day smoking period (Fig. 8C,D). In addition, IAV infection didn’t really impact on food intake nor did treatment with ebselen.

## Discussion

The primary aim of this study was to determine whether targeting oxidative stress with apocynin and ebselen could reduce lung inflammation in mice exposed to CS and infected with IAV, using an *in vivo* model that mimics AECOPD. We found that apocynin and ebselen generally reduced BALF inflammation, whole lung pro-inflammatory mediators and proteases, that ebselen reduced the amount of virus in the lungs but that neither apocynin nor ebselen affected CS and IAV-induced weight loss or food intake.

In the present study we found that CS-exposed mice had increased BALF total cells, macrophages and neutrophils compared to sham-treated mice. IAV infection alone also caused an increase in BALF macrophages and neutrophils but this was further amplified with prior CS exposure. These observations are consistent with previous studies showing that CS exacerbates the inflammatory response to IAV infection^10^,^37^.

We then went on to explore the mechanisms responsible for this increased BALF inflammation in CS + IAV mice. CS contains high levels of oxidants, which can increase BALF cellularity through cell recruitment, increased cell proliferation, and by prolonging cell survival in the airways^38^,^39^. Influenza A infection also causes the production of oxidants which further add to the oxidative burden induced by CS. Oxidants then activate redox sensitive transcription factors (such as NF-κB and AP-1) which lead to an increase in gene expression of pro-inflammatory cytokines and chemokines which are responsible for cell recruitment, survival and proliferation^40^. Since pro-inflammatory chemokine, cytokines and proteases regulate lung inflammation, we examined the expression of a wide range of pro-inflammatory mediators following CS exposure and influenza infection. CS exposure resulted in increased mRNA levels of cytokines (GM-CSF, TNF-α, IL-1β, IL-6), chemokines (CCL-2, CXCL-2, CXCL-10) and proteases (MMP-12). In addition, influenza infection in CS-exposed mice resulted in increased production of these pro-inflammatory cytokines, chemokines and proteases. Macrophages and neutrophils are increased in patients with COPD and further augmented following influenza infection^11^,^40^. Moreover, macrophages in lungs can be directly activated by CS to release inflammatory mediators such as CCL-2, which recruits monocytes from blood to increase the airway alveolar macrophage population^41^. Furthermore, activation and survival of neutrophils and macrophages are promoted by TNF-α and GM-CSF^35^,^36^,^42^. Therefore, the increase in CCL-2, TNF-α and GM-CSF in our study may explain the observed increase in macrophages and neutrophils following CS and influenza infection.

A potential limitation of our study is that we only measured mRNA levels of various cytokines, chemokines and proteases but did not measure protein levels of these mediators. Based on our previous study of BALF protein measurements in a similar model^10^, there was a poor correlation with mRNA levels and cytokine BALF levels in general were very low, as the increase in inflammation may cause increased consumption of BALF cytokines. We have previously published that CS exposure (without viral infection) was associated with higher protein levels of TNF-α, MIP-2, GM-CSF and IFN-γ in BALF than in no-smoke mice, especially at d10^10^. Also, IAV increased d3 protein levels of MIP-2 and MCP-1 more so with prior CS exposure. IAV infection alone did not increase other cytokines in BALF. However, when comparing IAV alone to CS and IAV mice, CS reduced the IAV-associated induction of mRNA encoding TNF-α, IL-1β, IL-6, and IP-10 at d3 and MIG (monokine induced by gamma-interferon) at d10. CS increased the IAV-associated induction of mRNA encoding IL-17. We also found that mRNA and protein levels for the same mediator were often not concordant; many of these inflammatory mediators may be subject to post-transcriptional regulation. Alternatively, given that mRNA and protein levels under certain conditions do not correlate then perhaps protein levels of the various mediators would be the better marker of inflammation and could likely contribute to the impaired immune response.

In the present study we found that CS + IAV mice had increased levels of lymphocytes compared to CS or IAV alone mice. This is consistent with findings in both animals^10^,^37^ and subjects with AECOPD^8^. It is probable that the observed increases in lymphocytes were due to increased CXCL-10 expression, a known chemotactic factor for lymphocytes. This is important as CD8^+^ T cells are critical for a protective memory response following influenza infection but when produced in excess can be involved in tissue damage in smokers^43^,^44^. Moreover, CD4^+^ T cells are involved in hypersecretion in smokers, which are known but not essential for clearance of influenza in mice^45^.

Neutrophils in COPD and AECOPD have the capacity to induce tissue damage through secretion of proteases^46^. In this study, there was an upregulation of matrix metalloproteinase 12 (MMP-12). The increased expression of MMP-12 in our study are in accord with COPD patients who have increased levels of MMP-12 in their lungs compared to healthy subjects without COPD^47^. Moreover, it has been shown that emphysema caused by chronic CS exposure is prevented in MMP-12^−/−^ mice^48^, suggesting that MMP-12 can have a destructive role in AECOPD. While our study focussed mainly on the impact of apocynin and ebselen on BALF inflammation, cytokine/chemokine/protease/ROS production, and viral load, it would be interesting to assess the effects of these compounds on chronic features of COPD (such as emphysema and changes in lung function) using models like those described by Beckett and colleagues^49^.

The increased BALF inflammation observed in our study may also be because existing mechanisms, such as production of endogenous anti-oxidants, to resolve lung inflammation are overwhelmed. Moreover, Robbins and colleagues showed that CS exposure with high dose of IAV infection increased the inflammatory response in lungs, in contrast to low dose of IAV infection, which resulted in decreased airway inflammation^11^.

Glutathione peroxidases (GPx) are cytosolic selenium-dependent and independent anti-oxidant enzymes that play a critical role maintaining and protecting bio-membranes against oxidative stress in various disease states^50^,^51^. Previous studies have shown that GPx-1 protects against CS and IAV (HKx31)-induced lung inflammation^29^,^30^. Moreover, the GPx mimetic ebselen has been shown to be protective *in vivo* in disease states hallmarked by oxidative stress such as diabetes-associated atherosclerosis, cerebral ischemia-reperfusion injury and lung inflammation^29^−^31^,^52^,^53^. In particular, ebselen reduces LPS-induced lung inflammation by reducing the levels of TNF-α and MIP-2, potent inducers of neutrophil activation^32^. In addition, ebselen has been used in clinical trials of acute ischemic stroke^54^,^55^. Specifically, Yamaguchi *et al.*^55^ explored the effects of ebselen on the outcome of acute ischemic stroke in a multi-center, placebo-controlled, double-blind clinical trial. They demonstrated that early treatment (i.e., patients who started ebselen within 24 h of stroke onset) with ebselen (150 mg bid) improved the outcome of acute ischemic stroke^55^. Similarly, Ogawa *et al.*^54^ showed in a randomized, double-blind, placebo-controlled trial of ebselen conducted in patients with complete occlusion of the middle cerebral artery that ebselen protected the brain from ischemic damage in the acute stage. More recently, ebselen has been trialled for prevention and treatment of noise-induced hearing loss (see ClinicalTrials.gov identifier: NCT01444846). Given the above, we expected that mice treated with ebselen would show a reduction in lung inflammation following CS and IAV infection. This was indeed the case and also attributed to its inhibitory effect on the various chemotactic factors and proteases involved in recruiting inflammatory cells into the lungs.

To further investigate the role of oxidative stress in this *in vivo* model of AECOPD, we used a pharmacological inhibitor of Nox-2, apocynin. Apocynin has been shown to have beneficial effects in a number of disease models including hypertension^56^, myocardial infarction^57^, asthma^58^ and influenza infections^18^. In addition, Orosz *et al*. has shown that apocynin can prevent CS-induced endothelial dysfunction and hinder the NADPH oxidase derived H_2_O_2_ generation in endothelial and smooth muscle cells^59^. The effects of apocynin on ROS production in human asthma and COPD have also been explored. Specifically, nebulized apocynin reduced the levels of H_2_O_2_ and nitrite (NO_2_^−^) ions in the EBC of COPD patients^34^. It has also been shown that nebulised apocynin significantly decreased the levels of H_2_O_2_, NO_2_^−^ and nitrate (NO_3_^−^) in EBC from asthmatic subjects^33^. These studies, and one performed in healthy subjects^60^, showed that apocynin is well tolerated and had no adverse events. Collectively, these studies suggest that apocynin may have potential for the treatment of bronchial asthma and COPD. Therefore, as anticipated, we found that apocynin reduced BALF cellularity, inflammatory gene expression and superoxide production in CS + IAV treated mice, highlighting the importance of redox-dependent inflammatory signalling in the lung.

Given that ROS are required for host defence against invading pathogens, it is conceivable that the suppression of ROS with pharmacological agents such as apocynin could increase susceptibility to secondary bacterial exacerbations. The evidence to support this possibility comes from studies of patients with a rare condition (1 in 200,000) known as chronic granulomatous disease (CGD). CGD patients have an ‘underactive’ ROS generating system in which their leukocytes are unable to mount a respiratory burst, leaving them susceptible to severe, life-threatening infections by opportunistic microbes such as bacteria and fungi^61^. CGD can be caused by several mechanisms including missense, nonsense, frameshift, splice and deletion mutations in the genes for Nox2, p22^phox^, p47^phox^, p67^phox^ or p40^phox^. Depending on the type of mutation, superoxide production may range from being ~0.1% to 10% of normal production^62^. A recent key study by Kuhns *et al.* provides evidence that CGD patients with mutations causing even modest residual ROS production (~1% of the normal amount) have a substantial survival benefit and antipathogen capabilities^62^. Indeed, an appropriate dosage of an inhibitor of Nox2 to reduce, rather than abolish, NADPH oxidase activity could be used feasibly without compromising the innate immune system in patients with IAV-induced lung oxidative stress and pathology. In addition, partial inhibition of ROS could be achieved by utilising inhibitors that target p47^phox^. Indeed, mutations in p47^phox^ - as observed in the study of Kuhns *et al.*^62^ - provide evidence that the oxidase is still capable of producing sufficient amounts of superoxide to serve anti-pathogen roles. In this study, and in our previously published work^18^, we have shown that apocynin decreases superoxide production in BALF inflammatory cells obtained from IAV-infected mice by approximately 30–50%. This is important as the remaining superoxide will serve anti-pathogen roles and should protect the host against susceptibility to bacterial exacerbations.

To determine if the exacerbated inflammatory response is due to greater viral replication and if the anti-oxidants have an effect in viral clearance, we assessed the amount of virus in the lungs. In accordance with our previous work^10^, sham + IAV mice had increased viral titres and this was further exacerbated in CS + IAV mice. However, apocynin did not affect viral load in lungs from IAV or CS + IAV mice. This is in contrast to our findings where apocynin reduced viral load in HKx31-infected mice^18^ suggesting that the effects of apocynin may depend on the strain of IAV used. In contrast to apocynin, CS + IAV mice treated with ebselen had significantly lower viral titres than CS + IAV + vehicle mice. This may be due to the increased levels of CXCL-10 and the anti-viral cytokine IFN-γ (data not shown) we observed in whole lungs of CS + IAV + ebselen mice compared with CS + IAV + vehicle mice. Furthermore, increased neutrophil numbers correlate with the enhanced viral clearance in IAV-infected mice^63^, but this was not the case in our study as neutrophils were reduced in ebselen-treated mice. This would suggest that ebselen potentially interfered with viral replication at a very early stage resulting in reduced cytokine production and reduced neutrophils. Another possibility is that virus specific CD8^ + ^T cells can play a role in clearance of influenza virus^10^, but these were not measured in the present study. Recently, Hsu *et al.* showed that the increased susceptibility of individuals with COPD to influenza likely results from impaired antiviral responses, which are mediated by increased PI3K-p110a activity^64^.

Cigarette smoke-mediated weight loss is well documented in the literature and this was confirmed in the present study. However, the combination of CS + IAV had no further effect on body weight nor did treatment with apocynin or ebselen. Increases in pro-inflammatory cytokines such as TNF-α have been observed in COPD and it could be that TNF-α regulates lipid metabolism by releasing leptin from adipose tissues^65^. This in turn acts on the neuropeptide Y in the hypothalamus to suppress appetite thereby leading to the weight loss^65^ observed in our study. Conversely, our findings that Mem71 flu infection did not affect body weight in mice is in accordance with other studies^10^, as it is of intermediate virulence despite its ability to induce a strong immune response. In addition, it would have been worth evaluating whether apocynin and ebselen influenced IAV-induced morbidity using a higher dose of Mem71 or alternatively using a more virulent strain of IAV such as A/PR/8/34^64^. This is important because if apocynin and ebselen improve the health of IAV-infected mice, then this would further add to the therapeutic potential of these compounds.

In conclusion, we found that in this animal model of CS and IAV (Mem71) infection, apocynin and ebselen reduced oxidative stress, the expression of pro-inflammatory mediators in the lung, BALF lung inflammation and viral titres but had no effect on CS and IAV-induced weight loss and food intake. The striking effects of apocynin and ebselen in our model suggest that they could be of important value in attenuating inflammation and improving viral clearance in AECOPD by targeting oxidative stress, either alone or when added to conventional therapy.

## Additional Information

**How to cite this article**: Oostwoud, L. C. *et al.* Apocynin and ebselen reduce influenza A virus-induced lung inflammation in cigarette smoke-exposed mice. *Sci. Rep.* **6**, 20983; doi: 10.1038/srep20983 (2016).

## Figures and Tables

**Table 1:** Effect of apocynin administration on whole lung cytokine, chemokine and protease mRNA expression in cigarette smoke (CS) and influenza A virus (IAV)-infected mice (3 days post infection).

Treatment								
Gene	Sham				CS			
	Dil + Veh	Dil + Apo	IAV + Veh	IAV + Apo	Dil + Veh	Dil + Apo	IAV + Veh	IAV + Apo
*Cytokines*								
GM-CSF	1.00	0.31	3.31	1.79	5.24	5.10	4.11	0.67
TNF-α	1.00	0.03	0.96	1.16	0.47	0.37	2.72	0.68
IL-1β	1.00	0.04	4.87	3.22	1.07	0.85	4.57	0.76
IL-6	1.00	0.04	15.89	14.89	0.43	0.85	24.58	4.51
Chemokines								
CCL-2	1.00	0.60	121.31	124.08	20.06	12.64	377.65	42.93
CXCL-2	1.00	0.15	15.97	7.09	6.77	5.36	23.98	4.21
CXCL-10	1.00	1.01	124.63	148.21	4.50	4.35	267.02	87.84
Proteases								
MMP-12	1.00	0.09	2.24	1.41	14.33	16.54	21.84	3.38

mRNA expression for all genes was measured simultaneously under identical conditions using quantitative real-time PCR. Responses for each time point are shown as fold change relative to sham + dil + veh mice after normalization to 18S rRNA (housekeeping gene). Data are shown as the average for duplicate reactions of 6–8 pooled whole lungs.

**Table 2:** Effect of apocynin administration on whole lung cytokine, chemokine and protease mRNA expression in cigarette smoke (CS) and influenza A virus (IAV)-infected mice (7 days post infection).

Treatment								
Gene	Sham				CS			
	Dil + Veh	Dil + Apo	IAV + Veh	IAV + Apo	Dil + Veh	Dil + Apo	IAV + Veh	IAV + Apo
*Cytokines*								
GM-CSF	1.00	2.20	1.44	1.20	1.44	1.72	2.80	1.45
TNF-α	1.00	6.33	38.19	27.17	4.58	6.48	135.90	34.67
IL-1β	1.00	0.24	3.6	1.58	1.96	3.06	36.60	18.57
IL-6	1.00	1.75	8.94	7.14	2.14	2.14	24.38	7.62
Chemokines								
CCL-2	1.00	3.02	109.74	84.04	3.85	2.7	132.1	94.8
CXCL-2	1.00	1.92	3.48	1.36	0.64	0.69	4.57	2.55
CXCL-10	1.00	0.55	96.53	44.79	0.88	0.53	64.41	49.69
Proteases								
MMP-12	1.00	2.73	18.55	8.19	31.04	46.23	74.69	39.72

mRNA expression for all genes was measured simultaneously under identical conditions using quantitative real-time PCR. Responses for each time point are shown as fold change relative to sham + dil + veh mice after normalization to 18S rRNA (housekeeping gene). Data are shown as the average for duplicate reactions of 6–8 pooled whole lungs.

**Table 3:** Effect of ebselen administration on whole lung cytokine, chemokine and protease mRNA expression in cigarette smoke (CS) and influenza A virus (IAV)-infected mice (3 days post infection).

Treatment								
Gene	Sham				CS			
	Dil + Veh	Dil + Ebs	IAV + Veh	IAV + Ebs	Dil + Veh	Dil + Ebs	IAV + Veh	IAV + Ebs
*Cytokines*								
GM-CSF	1.00	2.78	11.16	5.47	6.56	4.24	8.57	6.24
TNF-α	1.00	1.28	16.73	7.71	3.49	1.70	13.29	28.47
IL-1β	1.00	3.27	10.15	5.15	1.59	0.99	5.59	10.11
IL-6	1.00	2.53	295.99	123.56	5.46	7.52	128.63	98.91
Chemokines								
CCL-2	1.00	3.12	485.73	318.35	11.17	15.00	303.96	370.88
CXCL-2	1.00	1.91	59.45	41.18	8.44	7.42	31.57	38.96
CXCL-10	1.00	1.93	517.05	282.40	4.03	2.62	230.38	404.87
Proteases								
MMP-12	1.00	2.88	23.31	11.88	67.20	81.80	124.07	109.27

mRNA expression for all genes was measured simultaneously under identical conditions using quantitative real-time PCR. Responses for each time point are shown as fold change relative to sham + dil + veh mice after normalization to 18S rRNA (housekeeping gene). Data are shown as the average for duplicate reactions of 6–8 pooled whole lungs.

**Table 4:** Effect of ebselen administration on whole lung cytokine, chemokine and protease mRNA expression in cigarette smoke (CS) and influenza A virus (IAV)-infected mice (7 days post infection).

Treatment								
Gene	Sham				CS			
	Dil + Veh	Dil + Ebs	IAV + Veh	IAV + Ebs	Dil + Veh	Dil + Ebs	IAV + Veh	IAV + Ebs
*Cytokines*								
GM-CSF	1.00	1.14	2.71	2.14	0.07	0.07	12.32	9.71
TNF-α	1.00	0.92	2.19	3.12	0.32	0.36	20.50	12.82
IL-1β	1.00	0.94	1.05	1.36	0.31	0.30	7.92	5.67
IL-6	1.00	0.98	0.57	0.91	0.09	0.10	3.10	2.5
Chemokines								
CCL-2	1.00	1.14	2.71	2.14	0.07	0.07	12.32	9.71
CXCL-2	1.00	0.75	1.86	2.60	0.25	2.70	3.12	2.12
CXCL-10	1.00	1.05	14.75	27.12	0.24	0.37	153.53	122.86
Proteases								
MMP-12	1.00	0.84	0.44	0.05	1.34	1.27	14.59	8.87

mRNA expression for all genes was measured simultaneously under identical conditions using quantitative real-time PCR. Responses for each time point are shown as fold change relative to sham + dil + veh mice after normalization to 18 S rRNA (housekeeping gene). Data are shown as the average for duplicate reactions of 6–8 pooled whole lungs.
